# Anxiety and Depression Trajectories in Young Adults Up to 5 Years After Being Diagnosed With Cancer

**DOI:** 10.1002/cam4.70715

**Published:** 2025-03-06

**Authors:** Lars Sjödin, Sarah Marklund, Claudia Lampic, Lena Wettergren

**Affiliations:** ^1^ Department of Public Health and Caring Sciences Uppsala University Uppsala Sweden; ^2^ Department of Psychology Umeå University Umeå Sweden; ^3^ Department of Women's and Children's Health Karolinska Institutet Stockholm Sweden

**Keywords:** anxiety, cancer, depression, diagnosis, mental health, research, symptom, time, trajectory, young adult

## Abstract

**Aims:**

This study aimed to identify and characterize trajectories of anxiety and depression symptoms in a national cohort of young women and men up to 5 years after being diagnosed with cancer. Furthermore, potential sociodemographic, clinical, and psychosocial factors predictive of different trajectory groups were examined.

**Methods:**

A population‐based sample of 1010 young adults aged 18–39 at diagnosis with selected cancers/tumors (brain/breast/cervical/lymphoma/ovarian/testicular) completed a survey 1.5 years, 3 years (T2, *n* = 722) and 5 years (T3, *n* = 659) post‐diagnosis. Responses to the Hospital Anxiety and Depression Scale were computed using five trajectories as outcome groups: *Stable cases*, *Stable non‐cases*, *Improving*, *Worsening*, and *Fluctuating*. Multinomial logistic regression models were performed to identify predictive factors of different trajectories.

**Results:**

The most common trajectories for anxiety symptoms were Stable non‐cases (36%) and Stable cases (26%), followed by Improving (17%), Fluctuating (11%), and Worsening (10%). In contrast, the dominant trajectory for depression symptoms was Stable non‐cases (69%), with smaller groups identified as Improving (10%), Worsening (8%), Stable cases (7%), and Fluctuating (6%). Factors associated with several unfavorable trajectories were female sex, pre‐diagnosis support for emotional issues, fatigue, and financial problems (*p* < 0.05).

**Conclusion:**

Symptoms of anxiety and depression follow five different developmental paths among young people with cancer. Within the first 5 years after a cancer diagnosis, a majority of young adults meet clinical levels of anxiety (64%) and a third meet clinical levels of depression (31%). It is important to consider risk factors for mental illness in the follow‐up care of people with cancer.

## 1 Introduction

Advances in early detection and treatment of cancer, along with increased life expectancy, have resulted in a growing number of survivors in high‐income countries [1]. Mental health issues are more common among those with cancer than among those without cancer [2, 3, 4, 5], as well as among younger cancer survivors as opposed to their older counterparts [2]. Thus, monitoring mental health is especially important for young adults aged 18–40 [4, 6], as they have a potentially long life ahead of them.

Young adults who have experienced cancer constitute a particularly vulnerable group for mental health problems, as this age range concurs with crucial psychosocial development at risk of being disrupted [7, 8]. Establishing educational aspirations, financial resources, and social bonds with peer groups, partnerships, and family formation is distinctive for this life stage [9]. Additionally, young adulthood is a life stage when the onset of anxiety and depression disorders often takes place [10]. Cancer survivors may continue to struggle with emotional problems years after their treatment has ended. According to a recent systematic review, symptoms of depression in adolescent and young adult survivors appear to decrease with time, while the evidence on anxiety after a cancer diagnosis is less clear [2].

Studies have found that there are several factors linked with a higher risk of mental health problems among young adults who have been diagnosed with cancer. These factors include being female, being unmarried, being out of work or school, ongoing treatment, impaired physical function, experiencing pain, financial distress, and having poor family functioning [2, 11]. However, the risk factors for anxiety and depression among people diagnosed with cancer are relatively poorly characterized, and there is a lack of understanding of the long‐term mental health in this group [3, 4].

The bulk of evidence points to a knowledge gap warranting prospective longitudinal studies exploring change over time, as most research on mental health after a cancer diagnosis has been conducted on cross‐sectional data with small clinical samples [2, 3, 9, 12]. Relatively few studies have focused on young people's mental health following cancer [6, 13]. It is also pivotal to include various cancer types, as most studies have focused on breast cancer [6]. Much of the previous research has been qualitative, and scholars have called for studies using validated, standardized, and consistent measures used over time [2, 3, 13]. Studies exploring risk and protective factors related to mental health among young people with cancer are highly warranted [2, 6]. Given the lack of evidence on the long‐term development of anxiety and depression, the present study aims to identify and characterize trajectories of anxiety and depression symptoms in young adults with cancer up to 5 years after diagnosis. This study will also examine sociodemographic, clinical, and psychosocial factors that may predict these trajectory groups.

## 2 Method

### 2.1 Sample

**FIGURE 1:**
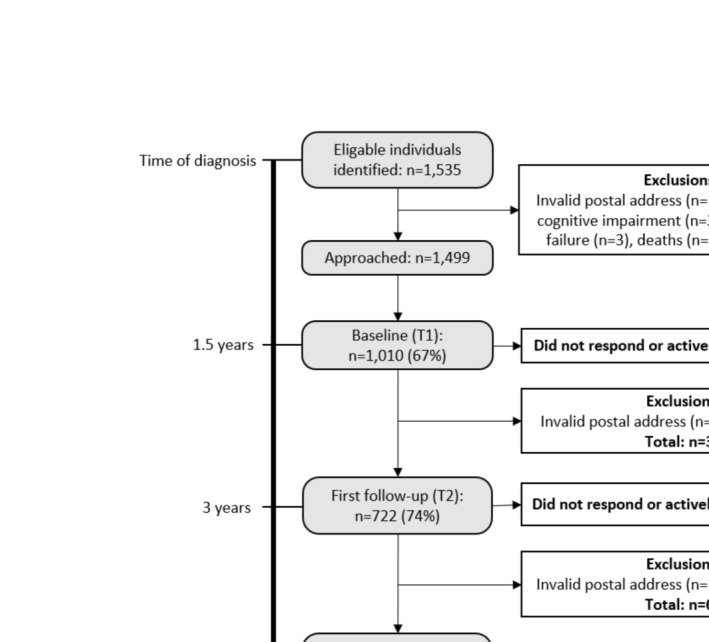
Flowchart of study participants.

This population‐based prospective cohort study has a longitudinal design following young adults up to 5 years post‐cancer diagnosis, as described in the study protocol [14]. The participants were asked to answer a self‐administered survey at three time points. Identification of eligible individuals was made via Swedish national quality registers. All individuals in Sweden diagnosed with selected cancers/tumors (brain/breast/cervical/lymphoma/ovarian/testicular) at age 18–39 from January 2016 to August 2017 were approached 1.5 years post‐diagnosis. Ethical approval for the study was granted by the Regional Ethical Review Board in Stockholm (record no: 2013/1746‐31/4; 2014/2244‐32; 2017/916‐32; 2017/1416–32). Written informed consent was collected from all participants before answering the survey. The number of study participants for each data collection is presented in Figure 1.

### 2.2 Variables

#### 2.2.1 Outcome

Anxiety and depression symptoms were measured using the Hospital Anxiety and Depression Scale (HADS) [15]. Each construct has seven items with responses corresponding to a score of 0–3 points. The composite total sum score ranges between 0 and 21 for each construct, with higher scores indicating higher levels of anxiety or depression. Cronbach's alpha was excellent at baseline for the anxiety scale (α = 0.85) and the depression scale (α = 0.81). A threshold of ≥ 8 points was used to differentiate levels of anxiety and depression with clinical relevancy (non‐cases 0–7 points, cases 8–21 points) [16].

**FIGURE 2:**
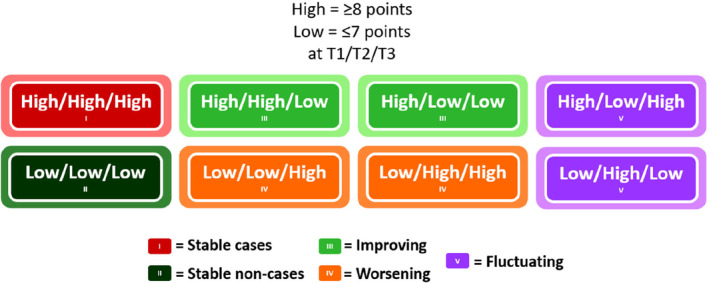
Trajectory groups by level of anxiety/depression symptoms at each time point.

Five trajectories were computed and used as outcome groups: *Stable cases* (≥ 8 points at T1, T2 and T3), *Stable non‐cases* (≤ 7 points at T1, T2 and T3), *Improving* (shifting from ≥ 8 points at T1/T2 to ≤ 7 points at T3), *Worsening* (shifting from ≤ 7 points at T1/T2 to ≥ 8 points at T3), and *Fluctuating* (alternating above and below the 8 point‐threshold at T1/T2/T3). The trajectory groups are illustrated in Figure 2.

#### 2.2.2 Predictors

Age (19–41), sex (male = 1/female = 2), and cancer treatment were retrieved from national quality registers. Healthcare professionals classified each individual's treatment data into four levels based on intensity (level 1–2/level 3–4) [17]. Self‐reported predictors at baseline were: birth country (Sweden = 1/other country = 2), education level (non‐university = 1/university = 2), main occupation (working or studying = 1/other = 2), partner (no = 0/yes = 1), having children (no = 0/yes = 1), and ongoing treatment, i.e., chemo, radiation, or other medical treatment (no = 0, yes = 1). Social functioning, fatigue, pain, and financial problems were measured at baseline using the subscales (0–100) of the quality‐of‐life instrument EORTC QLQ C‐30 [18]. Pre‐diagnosis support for emotional issues (no = 0, yes = 1) was self‐reported at T3.

### 2.3 Statistics

Analyses were performed using Stata/SE 18.5 and SPSS Statistics version 27. Mean values, proportions, and confidence intervals were used to describe characteristics and changes in symptoms of anxiety and depression within the sample and subgroups of interest. The *p*‐value for determining statistical significance was set to 0.05. Internal consistency was calculated using Cronbach's Alpha. Pearson's correlation test was applied to test for multicollinearity. Bivariable associations were explored, and significant predictor variables for either outcome were included in the multivariable analyses. Multinomial regression models were then performed to examine associations with groups of different trajectories in anxiety and depression. Stable non‐cases were used as reference groups for anxiety and depression, respectively.

## 3 Results

Characteristics of the sample at 1.5 years post‐diagnosis are presented in Table 1. Our sample was dominated by Swedish‐born women with a partner. Most participants had children, had higher education, and were currently studying or working. The most common diagnoses were breast and testicular cancer.

**TABLE 1:** Participant characteristics, by trajectory group and for total sample.

Variables	Improving group		Fluctuating group		Worsening group		Stable cases		Stable non‐cases		Total sample	
	Anx *n* = 98	Dep *n* = 53	Anx *n* = 61	Dep *n* = 35	Anx *n* = 55	Dep *n* = 45	Anx *n* = 147	Dep *n* = 40	Anx *n* = 199	Dep *n* = 387	Anx *n* = 560	Dep *n* = 560
Sociodemographic												
Sex Females, %	76.5	73.6	86.9	74.3	70.9	77.8	79.6	77.5	55.3	68.0	70.4	70.4
Age, mean	34.5	34.0	33.8	34.0	32.8	33.4	33.3	34.5	34.1	33.7	33.8	33.8
Birth country Sweden, %	91.8	83.0	82.0	82.9	85.5	80.0	83.7	85.0	89.4	89.1	87.1	87.1
Level of education University, %	62.2	56.6	68.9	77.1	61.8	66.7	59.2	42.5	60.8	62.3	61.6	61.6
Main occupation Working/studying, %	78.6	75.5	85.2	74.3	90.9	73.3	68.0	57.5	88.4	86.0	81.3	81.3
Children Having children, %	63.3	62.3	68.9	74.3	63.0	57.8	62.6	60.0	60.9	62.8	62.8	62.8
Partner In a relationship, %	85.6	83.0	83.6	80.0	81.8	75.6	81.0	75.0	85.9	86.3	83.9	83.9
Clinical												
Cancer diagnosis Breast, %	46.9	39.6	49.2	51.4	25.5	37.8	42.9	35.0	30.2	37.0	38.0	38.0
Cervical, %	14.3	22.6	16.4	8.6	29.1	13.3	16.3	12.5	14.1	17.1	16.4	16.4
Ovarian, %	4.1	5.7	—	2.9	5.5	2.2	4.1	5.0	2.5	2.8	3.2	3.2
Brain, %	13.3	5.7	11.5	11.4	10.9	15.6	11.6	22.5	15.1	12.9	13.0	13.0
Lymphoma, %	6.1	7.5	13.1	14.3	9.1	15.6	14.3	10.0	9.0	9.8	10.4	10.4
Testicular, %	15.3	18.9	9.8	11.4	20.0	15.6	10.9	15.0	29.1	20.4	18.9	18.9
Ongoing treatment Yes, %	43.9	22.6	36.1	51.4	18.2	28.9	32.7	20.0	25.6	31.8	31.1	31.1
Cancer treatment Most intensive, %	55.1	47.2	50.8	54.3	45.5	55.6	51.0	42.5	41.7	47.0	47.9	47.9
Pre‐diagnosis support for emotional issues Yes, %	33.7	41.5	39.3	54.3	32.7	44.4	47.6	60.0	16.6	24.0	31.8	31.8
Quality of life, 0–100												
Social functioning	65.8	56.6	69.9	62.4	90.0	69.3	58.6	41.3	88.4	82.5	74.8	74.8
Fatigue	45.9	60.4	38.8	52.2	28.9	43.2	53.5	64.4	25.6	30.6	38.2	38.2
Pain	25.5	33.0	22.4	31.0	16.7	25.9	32.5	42.1	12.1	16.2	21.4	21.4
Financial problems	17.0	24.5	20.8	30.5	11.5	23.0	32.4	51.7	5.9	10.4	17.0	17.0

The most common trajectory in anxiety symptoms was Stable non‐cases (36%), followed by Stable cases (26%), Improving (17%), Fluctuating (11%), and Worsening (10%). For trajectories in depression symptoms, the most common was Stable non‐cases (69%), followed by Improving (10%), Worsening (8%), Stable cases (7%), and Fluctuating (6%).

In Table 2, the changes in anxiety and depression mean scores are presented for each of the five trajectory groups. Despite the very different mean scores of anxiety and depression in the total groups, the development of the trajectory subgroups across the two conditions was rather similar. The Stable cases and Non‐cases groups did not change much across the study period. This consistency was also seen for both Fluctuating groups, although the pattern of change had different pathways for anxiety and depression in these groups. In contrast, the two Improving groups and the two Worsening groups underwent larger changes as their mean scores increased or decreased by almost five points.

**TABLE 2:** Mean score change in anxiety and depression symptoms by trajectory groups.

Variables	Improving group	Fluctuating group	Worsening group	Stable cases	Stable non‐cases	Total
Anxiety mean score at T1	9.9	8.2	5.4	12.7	3.5	7.7
Anxiety mean score at T2	6.8	7.5	7.9	12.5	3.1	7.2
Anxiety mean score at T3	5.2	8.4	9.9	12.0	3.3	7.1
Total change in anxiety means T1–T3	−4.7	+0.2	+4.5	−0.7	−0.2	−0.6
Observations in anxiety trajectory groups	98	61	55	147	199	560
Depression mean score T1	9.2	6.4	5.0	11.3	2.9	4.5
Depression mean score T2	6.1	7.7	6.4	11.2	2.6	4.2
Depression mean score T3	4.5	6.6	9.7	11.8	2.7	4.3
Total change in depression means T1–T3	−4.7	+0.2	+4.7	+0.5	−0.2	−0.2
Observations in depression trajectory groups	53	35	45	40	387	560

Predictors for the different trajectories in anxiety symptoms are presented in Table 3. Five factors significantly predicted belonging to another trajectory than the Stable non‐cases group. Female sex was related to an increased probability of unfavorable trajectories in anxiety when using Stable non‐cases as the reference. Participants with a self‐reported history of support for emotional issues and those reporting financial problems at baseline also had an increased probability of an unfavorable trajectory in anxiety. Reporting fatigue was positively related to both the group of Stable cases with consistently high anxiety and the Improving group, who only had high anxiety at baseline but later experienced a lower level. Furthermore, social functioning was negatively associated with several unfavorable trajectories.

**TABLE 3:** Factors associated with different trajectories in anxiety (*n* = 560). Stable non‐cases (*n* = 199) were used as the reference category.

Predictors	Improving group	Fluctuating group	Worsening group	Stable cases
Sex	1.85 (1.02–3.36)*	3.91 (1.71–8.97)*	1.78 (0.90–3.52)	1.85 (1.04–3.27)*
Level of education	0.97 (0.56–1.69)	1.18 (0.61–2.32)	0.88 (0.46–1.70)	1.00 (0.59–1.71)
Main occupation	1.07 (0.52–2.21)	0.67 (0.27–1.69)	0.60 (0.21–1.72)	1.27 (0.64–2.49)
Treatment intensity	1.24 (0.73–2.10)	0.99 (0.53–1.83)	1.04 (0.56–1.95)	0.97 (0.58–1.61)
Social functioning	0.98 (0.97–0.99)*	0.98 (0.97–1.00)*	1.02 (1.00–1.04)*	0.98 (0.97–1.00)*
Fatigue	1.02 (1.00–1.03)*	1.01 (0.99–1.02)	1.00 (0.99–1.02)	1.02 (1.01–1.04)*
Pain	1.00 (0.99–1.02)	1.00 (0.99–1.02)	1.01 (0.99–1.03)	1.01 (0.99–1.02)
Financial problems	1.01 (0.99–1.02)	1.02 (1.00–1.03)*	1.02 (1.00–1.03)*	1.02 (1.01–1.03)*
Pre‐diagnosis support for emotional issues	1.80 (0.98–3.31)	2.26 (1.15–4.43)*	2.20 (1.09–4.43)*	2.91 (1.66–5.11)*
Observations	98	61	55	147

*Note:* Results from a multinomial logistic regression model. Estimates are presented in risk ratios and 95% confidence intervals.

* Indicates significant difference (*p* < 0.05) compared with Stable non‐cases.

Predictors for groups with different trajectories in depression symptoms are presented in Table 4. Participants with a history of support for emotional issues were up to four times as likely to have an unfavorable trajectory in depression when using Stable non‐cases as the reference. Furthermore, experiencing fatigue or financial problems at baseline increased the risk of belonging to an unfavorable trajectory.

**TABLE 4:** Factors associated with different trajectories in depression (*n* = 560). Stable non‐cases (*n* = 387) were used as the reference category.

Predictors	Improving group	Fluctuating group	Worsening group	Stable cases
Sex (female)	0.75 (0.36–1.57)	0.62 (0.26–1.47)	1.04 (0.48–2.28)	0.65 (0.26–1.67)
Level of education	0.86 (0.44–1.68)	2.60 (1.05–6.43)*	1.24 (0.62–2.50)	0.54 0.23–1.23)
Main occupation	0.89 (0.40–1.98)	1.00 (0.40–2.50)	1.42 (0.65–3.12)	1.16 (0.48–2.84)
Treatment intensity	0.69 (0.37–1.32)	0.97 (0.46–2.03)	1.11 (0.58–2.12)	0.52 (0.24–1.14)
Social functioning	0.99 (0.98–1.00)	1.00 (0.98–1.01)	1.00 (0.98–1.01)	0.98 (0.96–0.99)*
Fatigue	1.04 (1.02–1.06)*	1.03 (1.01–1.05)*	1.01 (0.99–1.03)	1.03 (1.01–1.05)*
Pain	1.00 (0.99–1.01)	1.00 (0.98–1.02)	1.00 (0.99–1.02)	1.00 (0.99–1.02)
Financial problems	1.00 (0.99–1.02)	1.02 (1.00–1.03)*	1.01 (1.00–1.02)	1.02 (1.01–1.03)*
Pre‐diagnosis support for emotional issues	1.78 (0.91–3.47)	2.81 (1.32–5.97)*	2.09 (1.08–4.06)*	3.78 (1.68–8.48)*
Observations	53	35	45	40

*Note:* Results from a multinomial logistic regression model. Estimates are presented in risk ratios and 95% confidence intervals.

* Indicates significant difference (*p* < 0.05) compared with Stable non‐cases.

## 4 Discussion

This study aimed to identify and characterize groups with different trajectories of anxiety and depression symptoms in a sample of young adults with cancer up to 5 years after diagnosis.

We observed that symptoms of anxiety and depression followed five different developmental paths. The largest trajectory group for both anxiety and depression was Stable non‐cases. All remaining trajectory groups experienced clinical levels of symptoms on at least one occasion within 5 years after diagnosis. About 6 out of 10 met clinical levels of anxiety at some point, while the corresponding share was 3 out of 10 for depression. Individuals with unfavorable trajectories were more likely to be female, having received pre‐diagnosis support for emotional issues, as well as having financial problems and reporting fatigue at 1.5 years after diagnosis.

We found elevated levels of anxiety to be twice as common as depression, in line with other findings, showing even higher discrepancies [19]. This demonstrates that anxiety problems are a larger issue than depression among young adults with cancer. Despite highly different levels of anxiety and depression, the development of mean score symptoms of the trajectory subgroups across the two conditions was rather similar. This indicates that the differences in the prevalence of symptoms likely are driven by depression being the rarer condition rather than that the development of these conditions differs.

We have identified five different trajectories that demonstrate long‐term patterns in anxiety and depression symptom development among young adults after being diagnosed with cancer. Four of these trajectories were unfavorable, as they passed clinically relevant levels at some point during the study period. In other longitudinal studies on cancer patients, similar patterns in trajectories have been identified [20, 21, 22, 23, 24, 25]. Most have found four groups—one with *consistently low* symptoms, one with *consistently high* symptoms, one with *increasing* symptoms, and one with *decreasing* symptoms. Few have identified a fluctuating trajectory, and as of date, we have only found one other study describing this particular pattern [26]. The fluctuating symptoms of this group demonstrate that the need for support can wax and wane over time, underscoring the importance of continuous and responsive follow‐up care.

The mean scores of anxiety and depression symptoms in our study exceed the normal values observed among young adults in Germany, especially considering anxiety [27, 28]. This confirms that mental health problems are more common among people with cancer, as suggested by additional studies [2, 3, 4, 6]. However, the mean scores in our sample seem to be slightly higher for anxiety and lower for depression compared to other young adults with cancer [28]. We also found that mean scores of symptoms decreased slightly for the total group between 1.5 years, 3 years, and 5 years after diagnosis. However, the reductions were small, indicating that elevated levels of anxiety are a long‐term problem for this group. This adds evidence to previous findings of a systematic review reporting that depression among young adults with cancer generally decreases with time but that the studies on anxiety are less clear [2].

Several factors were able to differentiate subgroups with varying trajectories in anxiety and depression symptoms over 5 years after diagnosis. First, it is well known that females are particularly vulnerable to anxiety and depression [29], also among cancer survivors [30]. In our findings, sex was a central factor in the development of anxiety but not for depression.

To our understanding, an absent influence of sex on the development of depression symptoms among young adult cancer survivors is less known, as there is solid evidence of women generally being more likely to be depressed [31]. However, this finding may—in accordance with study results on advanced cancer patients [32] —suggest that the impact of cancer on depression trajectories affects women and men in young adulthood in a similar way. Second, we found that the experience of pre‐diagnosis support for emotional issues increased the risk of an unfavorable trajectory in anxiety and depression. This factor may serve as a proxy for a history of mental health problems, which previously have been found to influence anxiety and depression in cancer patients [33]. Third, financial problems were shown to be a crucial factor in predicting long‐term pathways in anxiety and depression symptoms. Financial hardship is more prevalent among young cancer survivors [34] and has also been shown to be closely linked to mental health [35].

The main strength of the present study is the large population‐based sample, including men and women identified through national quality registries containing clinical data. We expand previous research that has predominantly focused on breast cancer [6] by involving six common cancer diagnoses within this age group. Furthermore, to increase the reliability, we have used standardized measures psychometrically evaluated in people with cancer. The longitudinal design allowed us to examine continuity and change in symptoms. Longitudinal studies are often affected by attrition, resulting in skewed representation. In our study, substantially fewer males participated due to a smaller pool of eligible candidates for the selected diagnoses and lower response rates [36]. Despite this limitation, the overall generalizability is relatively high as the response rate was good throughout all time points. An additional limitation is that some of the trajectories resulted in small groups, possibly limiting power. Finally, as perceived social support has been linked to anxiety and depression among young adults with and without cancer diagnoses [37, 38], this variable should be included in future research of trajectories in this group of cancer patients.

Our findings underscore the need to develop routines to identify vulnerable groups of young adults with cancer not only during but also after treatment. To alleviate suffering and facilitate recovery, healthcare needs to provide support and treatment to match the mental health needs of this group. Financial problems and previous needs of mental support are important risk factors to consider in the rehabilitation of young adult cancer patients.

## 5 Conclusion

Symptoms of anxiety and depression follow five different developmental paths among young people with cancer. Within the first 5 years after a cancer diagnosis, a majority of young adults meet clinical levels of anxiety (64%) and a third meet clinical levels of depression (31%). It is important to consider risk factors for mental illness in the follow‐up care of people with cancer.

## Author Contributions

**Lars Sjödin:** conceptualization (equal), formal analysis (equal), methodology (lead), project administration (lead), writing – original draft (lead), writing – review and editing (equal). **Sarah Marklund:** formal analysis (equal), writing – original draft (equal), writing – review and editing (equal). **Claudia Lampic:** conceptualization (equal), funding acquisition (equal), methodology (supporting), resources (equal), supervision (supporting), writing – review and editing (equal). **Lena Wettergren:** conceptualization (lead), funding acquisition (equal), methodology (lead), project administration (supporting), resources (equal), supervision (lead), writing – review and editing (equal).

## Conflicts of Interest

The authors declare no conflicts of interest.

## Data Availability

The data that support the findings of this study are available from the corresponding author upon reasonable request.
